# Prevalence of DSM-IV TR Psychiatric Disorders in Children and Adolescents of Paveh, a Western City of Iran

**DOI:** 10.5812/ircmj.16743

**Published:** 2014-07-05

**Authors:** Nasrin Dodangi, Nastaran Habibi Ashtiani, Burhanoddin Valadbeigi

**Affiliations:** 1 Department of Psychiatry, Razi Hospital, University of Social Welfare and Rehabilitation Sciences, Tehran, IR Iran; 2 Health Center, Culham Construction Company, Paveh, IR Iran

**Keywords:** Adolescent, Child, Prevalence

## Abstract

**Background:**

Epidemiology, the study of patterns of disease distribution in time and space, can help to improve mental health services for children and adolescents by increasing understanding of causes, development, and course of psychiatric disorders**.**

**Objectives:**

To describe the prevalence of DSM-IV TR psychiatric disorders and comorbidities in students of Paveh, one of the western cities in Iran.

**Materials and Methods:**

The participants of this cross sectional survey were 379 children and adolescents aged 6-18 years old that were selected by multistage cluster sampling method. They were screened in the first phase of the study by two screening tools. In the second phase, 141 students were assessed by K-SADS-PL psychiatric interview.

**Results:**

The overall prevalence of DSM-IV TR disorders in this population according to psychiatric interview was 24.4%. The most common disorder was attention deficit hyperactivity disorder (11.9%) and then generalized anxiety disorder (11.3%), social phobia (6.2%), and separation anxiety disorder (6.2%). There was no significant difference between two sex and age groups except enuresis.

**Conclusions:**

The prevalence of psychiatric disorders in Paveh is comparable to other areas of Iran and the world. The high prevalence of attention deficit hyperactivity disorder needs more consideration and treatment plans.

## 1. Background

Epidemiology, the study of patterns of disease distribution in time and space, can help to improve mental health services for children and adolescents by increasing understanding of causes, development, and course of psychiatric disorders (1). Psychological community studies are helpful in evaluating the socio-demographic correlates of mental disorders in a given community (2). Social, cultural, political, and economical changes may influence physical and mental health in children and adolescents (3). Although it is difficult to get accurate estimates of child mental disorders, the few available epidemiological data indicates that 12-51%; with the average around 29% of the world’s children suffer from emotional and other mental problems that warrant mental health treatment (4). According to the World Health Organization (WHO), mental health disorders are one of the leading causes of disability worldwide (5). Three of the ten leading causes of disability in people between the ages of 15 and 44 are mental disorders, and the other causes are often associated with mental disorders. Both retrospective and prospective research has shown that most adulthood mental disorders begin in childhood and adolescence (6). Millions of children do not receive mental health services, are poorly bonded to supportive educational communities, and fail to develop important social and emotional competencies. About 7.5 million children have an unmet mental health need and only 6% to 8% of U.S. children receive adequate mental health services (7).

## 2. Objectives

The current study aimed to determine the prevalence rate of emotional and behavioral problems among Iranian children and adolescents in one of the western cities named Paveh.

## 3. Materials and Methods

### 3.1. Participants and Study Design

In this cross sectional study among the 5600 students in Paveh, 379 male and female students from all grades/levels were selected using the multistage cluster sampling method. In details, first we provided the list of all schools in Paveh from didactic administration. Since there were no significant cultural and socio-economic differences between various areas of Paveh, 10 schools from all levels were randomly selected proportional to the population size. Then considering sex, grade and level, some classes were randomly selected among these 10 schools. After that, the sample size was randomly selected among these classes.

The sample size was calculated using Cochran formula, as follows:

n=z2pqNd2(N-1)+z2 pq

N = Statistical population size: All students of Paveh were 5600

n = Sample size

Z = Standard normal variable, which is 1.96 at 95% confidence interval.

p = Ratio of a trait in the population, which can be considered as 0.5 if not available.

q = Percentage of those without that trait in the population (q = 1-p)

d = Acceptable margin of error, which is considered as 0.05

In the first phase of the research, parents of these 379 students were invited to attend to schools for participation in the study. Ethical issues that we considered included: to explain about the research and its benefits, but no coercion for participating and also explaining about confidentiality of gathered information. We also mentioned that they can leave the survey any time during the study.

After obtaining a written consent from the parents, they were asked to answer the general information questionnaire and the CSI-4 and CBCL screening questionnaires. Parents who refuse to cooperate were substituted with candidates from the same cluster. This phase was conducted by a trained team of two psychologists, one social worker; and one physician supervised the team. Next, the screening questionnaires were interpreted and cases scoring more than the cut off points and wit suspected psychiatric disorders (n = 191) were identified.

In the second phase of the research, all of these 191 students and their parents/guardians were invited to a clinic for psychiatric evaluation. They were interviewed by a child and adolescent psychiatrists using the semi-structured K-SADS-PL interview. Since the K-SADS-PL is not a diagnostic instrument designed for autism spectrum disorders, cases whose score was higher than the cut-off point in the autism scale in CSI-4 questionnaire were both subjected to K-SADS-PL and ADI-R interviews. This study was conducted in Paveh, Iran in 2012 and 2013.

### 3.2. Instruments

#### 3.2.1. Questionnaire of Demographic and Past History Information

This questionnaire include: age, sex, past medical and psychiatric history, development and educational status and perinatal problems. It also contains some information of family such as siblings and their order, age and education of the parents. This questionnaire was constructed by authors.

#### 3.2.2. Children’s Symptom Inventory (CSI-4)-Parent Form

It’s a scale based on DSM-TV diagnostic criteria for multiple disorders. The symptom count procedure is more commonly used and allows the clinicians to identify whether the child or adolescent is currently exhibiting the sufficient number of clinically relevant symptoms necessary for DSM-IV diagnosis (8). Validity and reliability of the parent form of the questionnaire were assessed in Iranian children and results showed appropriate validity and reliability (9).

#### 3.2.3. The Child Behavior Checklist (CBCL)

CBCL has been the gold standard for research and clinical work among broad-band behavior rating scales since its development in 1960s (10). CBCL include subscale scores for several specific areas, as well as composite scores for Internalizing, Externalizing and Total problems (8). Psychometric properties of the parent and teacher rating form of CBCL were investigated in an Iranian community sample. The results showed that CBCL is a useful instrument to consider ADHD and any disorders in community samples (11).

#### 3.2.4. The Schedule for Affective Disorders and Schizophrenia (K-SADS)

A semi-structured interview that allow the interviewer to make judgments during the interview (8). The reliability and validity of the Persian translation of K-SADS-PL were established in Iran before (12, 13).

#### 3.2.5. Autism Diagnostic Interview-Revised (ADI-R)

The ADI-R is a semi-structured interview of the child’s primary caretaker that includes many items pertaining to social communication. The diagnosis is reached using an algorithm based on scores on clusters of items that correspond to social relationships; verbal and nonverbal communication; and restrictive and repetitive, and stereotyped patterns of behavior (8).

### 3.3. Statistical Analysis

All variables and data were included in SPSS software version 18 and analyzed. Variables included sex, age group (6-11 and 12-18 years), siblings, birth order, past medical, developmental and psychiatric history, age and education of parents, pregnancy complications, delivery type, birth weight (< 2500 and ≥ 2500 g), family status (to be nuclear or not). X^2^ (chi square) and fisher exact test were used for evaluation of correlation between variables and disorders. Statistical significance was considered as P ≤ 0.05.

## 4. Results

Among a total of 379 students who screened, 52.8% were male and 47.2% were female. Mean age of students was 12.42 ± 3.57. 39.8% of them were 6-11 years and 60.2% were 12-18. Eight persons were excluded because their questionnaires were incomplete; therefore we interpreted questionnaires of 371 persons that of them, 190 screen-positive individuals (in one or both screening questionnaires) were invited to the interview, but 141 persons of them were attended. The interview was conducted by K-SADS-PL in order to determine the prevalence of their psychiatric disorders based on DSM-IV-TR criteria (Table 1). Given that K-SADS-PL cannot diagnose autism spectrum disorders, 16 students who according to the CSI-4 were likely to be diagnosed with these disorders, were interviewed using the autism diagnostic interview-revised (ADI-R) in order to confirm or reject this diagnosis.

In general, 8.4% of students in Paveh suffered from one disorder based on DSM-IV-TR criteria and 17.8% suffered from comorbidity (more than one disorder). The most common disorder was attention-deficit hyperactivity disorder (11.9%) and then generalized anxiety disorder (11.3%), social phobia (6.2%), and oppositional defiant disorder (6.2%). Table 2 shows the overall results of prevalence and its differences in boys and girls. Also, prevalence in two age groups was shown in Table 3.

Since a number of students who had disorders in the primary screening test were not cooperative in conducting the diagnostic interview, their demographic information and the results of their screening tests were compared with that of the group undergoing to interview. There was no significant difference between these two groups in terms of their demographic characteristics such as age, sex, educational status, parents’ age and education level, history of prenatal and birth problems, history of physical illness, and developmental status. There was a significant difference between the non-cooperative group and interviewed group on incidence of specific phobia (x^2^ = 7.21, df = 1, P = 0.007) and obsessive compulsive disorder (x^2^ = 5.66, df = 1, P = 0.017) in CSI-4. In other words, if the first group could undergo for psychiatric interview, the prevalence of these two disorders (specific phobia and obsessive compulsive disorder) could have been much higher. However, the prevalence of other disorders probably would not have been significantly different.

**Table 1.** Total Number and Percentage of Participants in Both Sexes and Age Groups^a^

	Negative Screening	Positive Screening	Uncooperative for Interview	No Disorder on Interview	With One Disorder	With More than One Disorder	Total
**Age, y**							
12-18	113 (62.4)	113 (37.6)	34 (69.4)	19 (56.8)	19 (61.3)	41 (62.1)	226 (60.9)
6-11	68 (37.6)	77 (62.4)	15 (30.6)	25 (43.2)	12 (38.7)	25 (37.9)	145 (39.1)
**Sex**							
Female	84 (46.4)	92 (53.6)	21 (42.9)	23 (52.3)	15 (51.6)	33 (50)	176 (47.4)
Male	97 (53.6)	98 (46.4)	28 (57.1)	21 (47.7)	16 (48.4)	33 (50)	195 (52.6)
**Total**	181 (48.8)	190 (51.2)	49 (13.2)	44 (11.9)	31 (8.4)	66 (17.8)	371 (100)

^a^Data are presented as No. (%).

**Table 2.** Prevalence and Differences of Psychiatric Disorders According to DSM-IV TR in Two Sexes

	Sex, No. (%)		Fisher			X^2^		Total, No. (%)
	Female	Male	Degree Freedom	P Value	f	Degree Freedom	P Value	
**Attention deficit hyperactivity disorder**	15 (8.5)	29 (14.9)	3	0.051	2.612	3	0.051	44 (11.9)
**Major depressive disorder**	10 (5.7)	7 (3.6)	3	0.701	0.474	3	0.698	17 (4.6)
**Dysthymia**	2 (1.1)	1 (0.5)	3	0.762	0.384	3	0.760	3 (0.8)
**Adjustment disorder (depression)**	1 (0.6)	0	3	0.609	0.609	3	0.607	1 (0.3)
**Social phobia**	12 (6.8)	11 (5.6)	3	0.832	0.291	3	0.830	23 (6.2)
**Specific phobia**	7 (4.0)	2 (1.0)	3	0.285	1.267	3	0.284	9 (2.4)
**Generalized anxiety disorder**	26 (14.8)	16 (8.2)	3	0.240	1.407	3	0.239	42 (11.3)
**Obsessive compulsive disorder**	6 (3.4)	3 (1.5)	3	0.596	0.630	3	0.594	9 (2.4)
**Separation anxiety disorder**	4 (2.3)	2 (1.0)	3	0.679	0.506	3	0.676	6 (1.6)
**Enuresis**	4 (2.3)	4 (2.1)	3	0.844	0.274	3	0.842	8 (2.2)
**Encopresis**	2 (1.1)	0	3	0.416	0.951	3	0.413	2 (0.5)
**Oppositional defiant disorder**	9 (5.1)	14 (7.2)	3	0.549	0.706	3	0.546	23 (6.2)
**Bipolar I disorder**	1 (0.6)	0	3	0.609	0.609	3	0.607	1 (0.3)
**Bipolar II disorder**	2 (1.1)	0	3	0.416	0.951	3	0.413	2 (0.5)
**Chronic motor Tic**	1 (0.6)	2 (1.0)	3	0.762	0.387	3	0.760	3 (0.8)

**Table 3.** Prevalence and Differences of Psychiatric Disorders According to DSM-IV TR in Two Age Groups

	Total	Age, y		Fisher			X^2^	
		12-18	6-11	Degree Freedom	P Value	f	Degree Freedom	P Value
**Attention deficit hyperactivity disorder**	44 (11.9)	21 (9.3)	23 (15.9)	3	0.179	1.643	3	0.178
**Major depressive disorder**	17 (4.6)	13 (5.6)	4 (2.6)	3	0.105	2.060	3	0.105
**Dysthymia**	3 (0.8)	2 (0.9)	1 (0.7)	3	0.395	0.995	3	0.393
**Adjustment disorder (depression)**	1 (0.3)	1 (0.4)	0	3	0.302	1.220	3	0.300
**Social phobia**	23 (6.2)	11 (4.9)	12 (8.3)	3	0.299	1.229	3	0.297
**Specific phobia**	9 (2.4)	4 (1.8)	5 (3.4)	3	0.333	1.141	3	0.330
**Generalized anxiety disorder**	42 (11.3)	29 (12.8)	13 (9.0)	3	0.075	2.319	3	0.075
**Obsessive compulsive disorder**	9 (2.4)	5 (2.2)	4 (2.8)	3	0.417	0.950	3	0.414
**Separation anxiety disorder**	6 (1.6)	2 (0.9)	4 (2.8)	3	0.237	1.417	3	0.236
**Enuresis**	8 (2.2)	0	8 (5.5)	3	0.003	4.854	3	0.003
**Encopresis**	2 (0.5)	1 (0.4)	1 (0.7)	3	0.411	0.961	3	0.409
**Oppositional defiant disorder**	23 (6.2)	17 (7.5)	6 (4.1)	3	0.093	2.153	3	0.093
**Bipolar I disorder**	1 (0.3)	1 (0.4)	0	3	0.302	1.220	3	0.300
**Bipolar II disorder**	2 (0.5)	2 (0.9)	0	3	0.215	1.495	3	0.214
**Chronic motor Tic**	3 (0.8)	2 (0.9)	1 (0.7)	3	0.395	0.995	3	0.393

## 5. Discussion

Various studies on the prevalence of child and adolescent psychiatric disorders worldwide show various and contradictory reports. In a recent study in an eastern city of China, the estimated prevalence of behavioral problems was 10.5% based on the cutoff point for behavioral problems according to the CBCL (14). In Vietnam, the most commonly reported problem in children and adolescents was anxiety disorders, especially simple and social phobias (15). In a national study on the lifetime prevalence of psychiatric disorders among American adolescents the most common disorders were anxiety disorders (31.9%), behavioral disorders (19.1%), mood disorders (11.3%), and substance abuse disorders, respectively (16). In a study on children and adolescents aged 5-16 in Bangalore, India; the prevalence of psychiatric disorders was reported as 12.5%. Enuresis, specific phobia, hyperkinetic disorders, stuttering and oppositional defiant disorder were the most frequent diagnosis (17).

In another study in US on 9-17 years-old rural population of North Carolina, the prevalence of psychiatric disorders in African Americans and Caucasians was reported as 20.5% and 21.9%, respectively (18). In another study in Carolina, the three-month prevalence of any child and adolescent psychiatric disorder was obtained as 13.3% (19). In one study on male Saudi schoolchildren, 8.3% were emotionally and/or behaviorally disturbed student (20). In 1999, 10438 British children and adolescents were assessed by clinicians. The overall prevalence of DSM-IV disorders was 9.5% (21). In a national representative survey in Latin America, the prevalence rate for any psychiatric disorder in Chile was 22.5% and this rate was higher among the children in comparison with adolescents (22). A systematic review indicated that 15% of children and youth have clinically important mental disorders if measures of impairment are included. Nevertheless, this prevalence rate is still high (23).

Several studies with a variety of tools and methods have also been conducted in different regions of Iran. One of them is the study of Mohammadi et al. in which using the SDQ screening questionnaire, they evaluated the prevalence of psychiatric disorders among adolescents in Tehran. Attention-deficit hyperactivity disorder, oppositional-defiant disorder, and separation anxiety disorder were the most commonly reported disorders among adolescents in Tehran (24). In another study conducted by Alavi et al. with a similar method on school children of Tehran, the most commonly reported disorders were attention deficit hyperactivity disorder (8.6%), oppositional defiant disorder (7.3%), and separation anxiety disorder (5.9%), respectively. In terms of gender differences, the prevalence of enuresis among males and anorexia nervosa among females was higher and there was no significant difference between the two genders in terms of the prevalence of other disorders (25). In our study, the prevalence of the disorders was not statistically different between girls and boys, and also between the age groups, except enuresis that was higher in children than adolescents. In the present study, the prevalence of depression, dysthymia, and anxiety disorders among females and the prevalence of attention-deficit hyperactivity disorder and oppositional defiant disorder among males were higher, which is consistent with the developmental course of the disorders; however this difference was not statistically significant.

As far as we know, this is the first study on child and adolescent psychiatric disorders in this population. According to the results, the prevalence of psychiatric disorders in this population was estimated to be approximately 24%, which is comparable to that of other areas in Iran, and relatively higher than some other countries. Moreover, the prevalence of attention-deficit hyperactivity disorder was estimated to be approximately 11.9%, which is almost higher than the prevalence of the disorder in most of the similar studies.

The present study has some strong points as follows:

1. On this study, two screening tools were simultaneously used, which reduced the number of false negative cases. That is why almost half of the samples screened underwent for clinical interview. This ratio is relatively high compared with other studies.
2. Given the statistical population size, the sample size was sufficient.

The present study has also some limitations or weak points as follows:

First, since this study has been conducted on students of ordinary schools, who usually have normal or high intelligence, it has not been able to estimate the prevalence of mental retardation.
Second, due to cultural reasons, the researchers have not been able to follow up the non-cooperative cases for interviewing.
Another limitation of the study is that since it has been based on parental reports, the prevalence of such disorders in some cases might have been over or under diagnosed.

Moreover, the presence or absence of demographic and physical risk factors were recorded and reported only by parents, which were likely to be biased. No autistic disorder was reported in the present study, which might be due to the fact that a large percentage of the children with autism spectrum disorder have low intelligence and will not naturally attend ordinary schools; and therefore, they were not included in the study. Also, we didn’t detect any conduct disorder, maybe it was due to absence of teacher’s information. As, in one study, a comparison of the disorders in children with and without teacher reports suggested that the prevalence of conduct disorders and ADHD would be underestimate in absence of teacher information (21). Furthermore, because of the cultural differences, we can’t generate these results to other cities of the country.

### 5.1. Human Subject Approval Statement

This study was approved by the Ethical Committee for Medical Sciences at University of Social Welfare and Rehabilitation Sciences in 17 November 2013. The ethic approval code is “USWR. REC. 1392.46”.
